# Reliability and cross-cultural validity of a Japanese version of the Dental Fear Survey

**DOI:** 10.1186/1472-6831-9-17

**Published:** 2009-07-10

**Authors:** Toshiko Yoshida, Peter Milgrom, Yukako Mori, Yukie Nakai, Mari Kaji, Tsutomu Shimono, Ana Nora A Donaldson

**Affiliations:** 1 Center of the Development of Medical and Healthcare Education, Okayama University, 2-5-1, Shikata-cho, Okayama City, Okayama, 700-8525, Japan; 2 Dental Fears Research Clinic, University of Washington, Box 357475 University of Washington, Seattle, WA 98195-7475, USA and the Department of Special Care Dentistry, King's College Dental Institute, London, UK; 3 Department of Behavioral Pediatric Dentistry, Okayama University Graduate School of Medicine, Dentistry and Pharmaceutical Sciences, 2-5-1, Shikata-cho, Okayama City, Okayama, 700-8525, Japan; 4 Biostatistics Unit, King's College Dental Institute, Cutcombe Road, Denmark Hill, London, UK

## Abstract

**Background:**

This study established the reliability and cross-cultural validity of a Japanese version of the Dental Fear Survey (DFS).

**Methods:**

Two studies were carried out in separate populations. The first involved 166 Japanese dental and nursing students and assessed internal consistency and test-retest reliability. The second involved 2,095 Japanese parents or guardians of school children and tested the hypothesis that the conceptual structure of the Japanese translation was consistent with the U.S. version using Structural Equation Modeling (SEM).

**Results:**

In the first study Cronbach alpha ranged from .94 to .96 and test-retest reliability (Spearman correlation) ranged from .89 to .92. The intra-class correlation coefficients (ICC) was 0.919 (95%CI: 0.892 – 0.940). In the second study SEM was used on the covariance matrix of the 20 questions in a random sample of 600 questionnaires to evaluate the goodness of fit of the theoretical model; and then, in an exploratory manner corrected for specification errors until a model that fit the data well was achieved.

**Conclusion:**

The Japanese version of the DFS appears reliable and demonstrates cross-cultural validity. The modeling confirms the three factors on which the English language version was based.

## Background

The Dental Fear Survey (DFS) was developed in the United States [1,2] and has been translated and used in many countries. A Japanese version was published but was never validated [3,4]. Convenience samples have been studied and fear levels reported have been extremely high. One study surveyed 415 Japanese university students: six to 14% scored 4 (very afraid) or 5 (terrified) on the DFS general item rating fear and 80% reported being a little afraid, somewhat afraid, very afraid or terrified of dental treatment on this measure. A second study surveyed 3,041 Japanese middle school students. Over 20% reported scores 4 or 5 on the general fear item. These very high levels of fear reported increase the need to understand the properties of the instrument being used across cultures and raise questions about the reliability and cross-cultural validity of the translated instrument.

Other Japanese researchers studied dental fear in 174 new patients (mean age 41 years, 36% male) at a dental hospital in Tokyo [5]. Dental fear was self-reported as part of a larger battery of assessments on a 100 mm visual analog scale anchored by "no fear" and "severe fear". Higher scores indicated greater fear. The overall mean score was 51.2 mm. Scores for fear were higher in males than females. Scores were correlated with the SF-36, which represents an individual's general health. Dental fear, satisfaction with tooth color, etc., which are considered psychological elements, were significantly correlated with several of the SF-36 subscales. The author suggested that psychological oral health elements affect the general status. Unfortunately the failure to use validated instrumentation in this study precludes knowing how these findings relate to fear in the larger population. Another research group studying a large sample of teenagers, and using yet another set of questions, found between 22 and 44% of the teens reported being fearful of dental checkups or treatment [6].

The aim of this study was to construct and psychometrically characterize a Japanese version of the DFS [See Additional file 1]. Two studies were undertaken. In study 1, the instrument was translated and its psychometric properties assessed. In study 2, the validity of the instrument was established. The DFS was designed to allow individuals to report three aspects of dental fear: avoidance, physiological arousal, and fear of specific situations at the dentist. It was hypothesized that the structure of the Japanese translation of the instrument would be consistent with the original US version [7].

## Methods 

### Study 1

#### Methods

##### Population

The participants were a convenience sample of 188 dental and nursing students of Okayama University. The students were told that participation was voluntary and that not participating would not result in any disadvantage in their grade in class. In case that they did not intend to participate, they were told to leave the questionnaire unanswered. Therefore, the students did not know who participated and who did not, reducing the potential for embarrassment. This is considered an educational activity and permitted under Okayama University rules and did not require review by the Ethics Committee.

##### Instrument

The DFS [7] was newly translated into Japanese because some of the previous Japanese translation was not comparable with the original English language version. The items most inconsistent with the English (see Table 1) dealt with avoidance (item 2) and specific stimuli (item 19). DFS was translated from English into Japanese by one of the researchers (YN), and then back translated by another researcher (TY) to ensure comparability with the original form. The DFS is made up of 20 items. Items are measured on a five-point Likert-like scale ranging from "not at all" (score 1) to "very much" (score 5). Total scores possible range from 20 to 100, where a higher score indicates greater fear.

**Table 1:** Mean DFS item scores*, study 1 and study 2

	Study 1 (The first administration) (n = 166)		Study 2 (n = 2095)	
Items	Mean	SD	Mean	SD

Avoidance				
1. Put off making appointment	1.4	0.8	1.5	0.9
2. Cancelled or failed to appear	1.1	0.4	1.2	0.6
8. Making an appointment	1.4	0.8	1.4	0.7
9. Approaching dental office	1.9	1.0	1.7	0.9
10. Sitting in the waiting room	1.8	1.0	1.7	0.9
11. Sitting in dental chair	2.1	1.0	2.0	1.0
12. Smell of dental office	1.8	1.0	1.6	0.9
13. Seeing the dentist	1.8	1.0	1.7	0.9
Physiological arousal				
3. Muscle tenseness	2.2	0.9	2.3	1.0
4. Increase breathing rate	1.5	0.7	1.8	0.9
5. Perspiration	1.6	0.8	1.6	0.8
6. Nausea	1.3	0.7	1.3	0.6
7. Heart beat faster	2.0	0.9	1.8	0.9
Fears of specific stimuli/situations				
14. Seeing anesthetic needle	2.7	1.3	2.5	1.2
15. Feeling anesthetic needle	2.6	1.3	2.5	1.2
16. Seeing drill	2.5	1.3	2.3	1.2
17. Hearing drill	2.6	1.4	2.5	1.3
18. Feeling drill	2.6	1.3	2.5	1.2
19. Having teeth cleaned	1.3	0.7	1.4	0.8
20. Overall fear of dentistry	2.1	1.0	2.1	1.0
Total	38.3	13.9	37.4	14.1

* Items are scored from 1 (not at all afraid) to 5 (terrified).

##### Procedure

The questionnaire was administered twice, one week apart. Participants did not have access to their earlier survey.

##### Analysis

The data were edited and entered into SPSS (version 14), and descriptive statistics calculated. Cronbach alpha was used to assess internal consistency and Spearman's Rank Correlation as well as intra-class correlation coefficient (ICC) was used to establish test-retest reliability.

#### Results

The questionnaire was administered in class and 169 students completed the DFS both times. Three questionnaires were not included in the analysis because of missing data. Of the 166 remaining subjects, 104 (63%) were female. Ages ranged from 18 to 37 years, with a mean age of 21.6 years (SD = 2.7).

The DFS means and standard deviations for the first and second time for the total sample were 38.3 (SD = 13.9), 36.6 (SD = 14.6), respectively. Cronbach alphas for the DFS were 0.94 and 0.96 for the first and second administrations, respectively. Spearman's Rank Correlation coefficients for test-retest reliability ranged from .89 to .92 (p < 0.001). The intra-class coefficient was 0.919 (95%CI: 0.892 – 0.940).

### Study 2

#### Methods

##### Population

The participants were 2,095 parents or guardians whose children were enrolled in six nursery schools, two kindergartens, and six public primary schools in Okayama and Kurashiki, Japan. The Ethics Committee of Okayama University Graduate School of Medicine, Dentistry and Pharmaceutical Sciences approved the study. The principals of each of the schools gave approval for the study. Participation was anonymous.

##### Instrument

The same Japanese version of the DFS developed for Study 1 was used in Study 2.

##### Procedure

School recruitment was done with the assistance of alumni of Okayama University Dental School. Potential participants could refuse to participate without any risk to benefits to which they were otherwise entitled. The confidentiality of personal data was assured in writing. Classroom teachers distributed the questionnaire. Parents or guardians completed the questionnaire at home.

##### Analysis

Cross-cultural validity was established by testing the hypothesis that the structure of the translated instrument was consistent with the original U.S. version (avoidance [items 1, 2 and 8–13], physiological arousal [items 3–7], and fears of specific situations [items 14–20]).

The survey data were entered into SPSS (version 14) for descriptive analyses and then imported into EQS software (Multivariate Software, Inc., Encino, CA) for SEM analysis. A random sample of 600 cases was chosen to avoid inflating p-values solely because of sample size.

SEM was used on the covariance matrix of the 20 questionnaire items to evaluate the goodness of fit of the theoretical model; and then, in an exploratory manner corrected for specification errors until a model that fit the data well was achieved. This involved eliminating parameters with small t-values (unless they had practical importance) and adding parameters with large modification indices if they were theoretically sound. Generalized least squares (GLS) and robust standard errors were used to validate the results.

## Results

The response rate was 81.0% (2,198/2,714). One-hundred-and-three questionnaires were eliminated from the analysis because one or more items on the questionnaire and/or demographic information such as age, gender, or the guardian's relationship to the child was left unanswered. Thus, the final sample consisted of 2,095 subjects with a mean age of 35.6 years, (SD = 4.7; range 22–66; 93% female).

**Figure 1:**
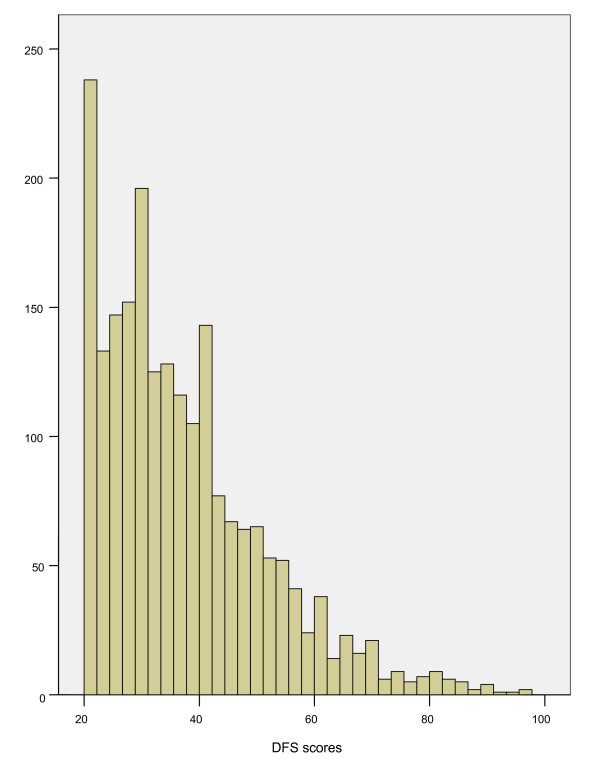
**The distribution of the total DFS scores (n = 2,095)**.

The DFS mean for this population was 37.4 (SD = 14.1). The range was from 20 to 96. The distribution of the DFS scores is given in Figure 1. The individual item scores are given in Table 1. The most fear-provoking items were the sight and feeling of the needle and the sound and feeling of the drill.

**Figure 2:**
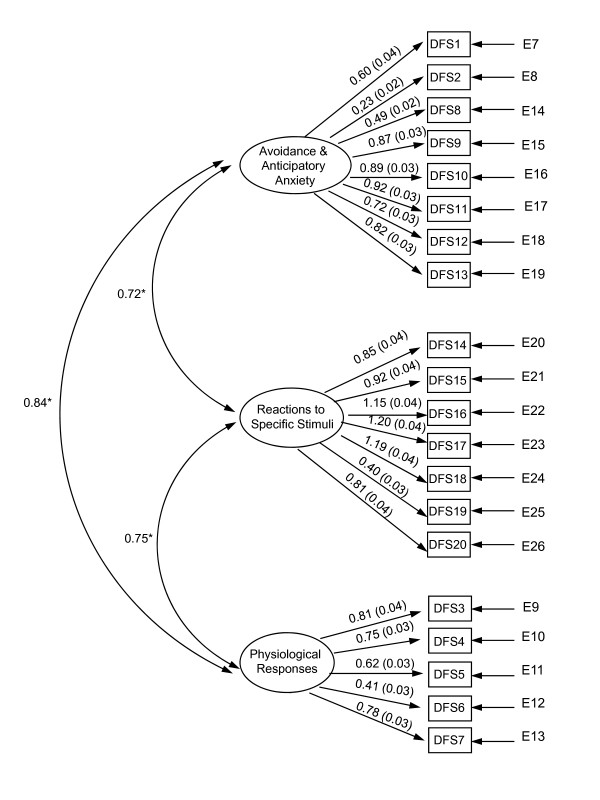
**Hypothesized model of the Dental Fear Survey based on the work of Kleinknecht et al. based on a random sample of 600 questionnaires**.

**Figure 3:**
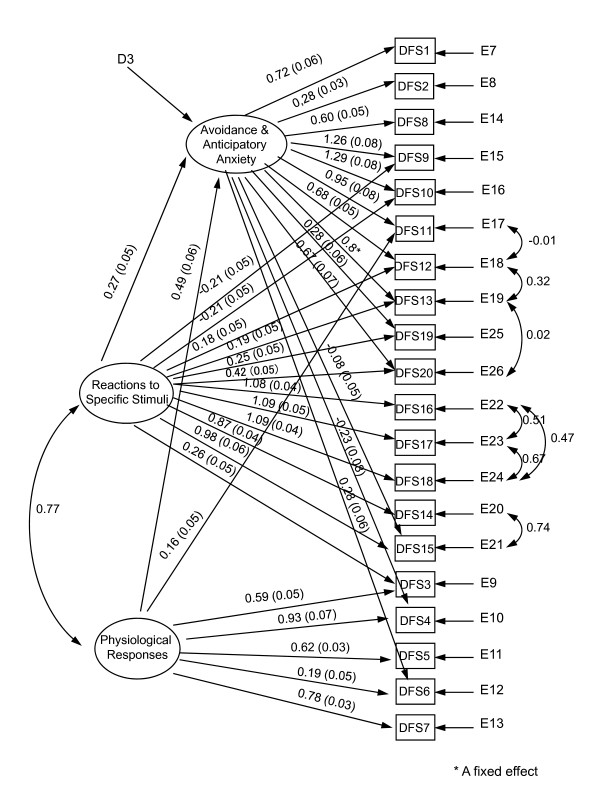
**Best fitting model of the Dental Fear Survey taking into consideration correlations between items based on a random sample of 600 questionnaires**.

Figures 2 and 3 illustrate the hypothesized and best fitting model, respectively. The goodness of fit test of the originally hypothesized structure resulted in Satorra-Bentler chi-square (df 167) = 1617, p < .00001 and the goodness of fit statistics were: root mean-square error of approximation RMSEA = 0.12 (95% Confidence Interval, CI: 0.11 – 0.13); comparative fit index CFI = 0.87; normed fit index NFI = 0.85 and the non-normed fit index NNFI = 0.85.

Although the fit indices of this hypothesized model were not poor, the Lagrange-Multiplier Test (LMtest) signalled some strong correlations between items which lead us to contemplate the model shown in Figure 3 (best fit model), with a goodness of fit chi-square (df 149) = 496, a considerable improvement in terms of the goodness of fit chi-square for the hypothesized model (a 70% reduction in the value of the statistic and a 10-fold reduction in its significance level). In addition, the fit indices of the best fit model reached the thresholds that qualify it as a good fit: well above 90% for AIC, NFI, NNFI and CFI and below 8% for the RMSEA.

Similar results were found using GLS and robust standard errors; the scaled chi-square, goodness of fit indices and the maximum likelihood estimators and their standard errors were essentially unchanged. The results were also confirmed when the model was applied to the overall sample of 2,095, showing similar goodness of fit statistics: a Satorra-Bentler chi-square (df 149) = 314; AIC = 16; NFI = 0.92; NNFI = 0.94; CFI = 0.96; RMSEA = 0.07 (0.06 to 0.09).

As shown in Figure 3, the latent factor Avoidance and Anticipatory Anxiety increases with each response level increase of: DFS1 by 0.72; DFS2 by 0.28; DFS8 by 0.60; DFS9 by 1.26; DFS10 by 1.29; DFS11 by 0.95; DFS12 by 0.68; DFS13 by 0.8; DFS19 by 0.28; DFS20 by 0.67; and DFS6 by 0.28. It decreases for each level increase of DFS4 by 0.23 and DFS15 by 0.08.

The latent factor Reactions to Specific Stimuli (Figure 3) increases with each level increase of: DFS12 by 0.18; DFS13 by 0.19; of DFS19 by 0.25, of DFS20 by 0.42, of DFS16 by 1.08, of DFS17 by 1.09, DFS18 by 1.09; DFS14 by 0.87; DFS15 by 0.98; and DFS3 by 0.26. It decreases for each level increase of DFS9 by 0.21 and DFS10 by 0.21.

The latent factor Physiological Responses (Figure 3) increases with each level increase of: DFS11 by 0.16; DFS3 by 0.59; DFS4 by 0.93; DFS5 by 0.62; DFS6 by 0.19; and DFS7 by 0.78.

## Discussion

### Reliability

The Japanese translation of the DFS showed good internal consistency and test-retest reliability, similar to that demonstrated by Kleinknecht and colleagues in assessing the original instrument in English [7].

### Cross-cultural Validity

The hypothesis that an acceptable model, embodying the original concepts in the English language instrument, could be fit to the Japanese version data was confirmed. Nevertheless, a more sophisticated model that takes into consideration logical correlations between items and the latent variables was consistent with the original model but explained the data better. Avoidance and fear of specific situations predict physiological responses and are associated with high levels of physiological responding and fear of specific situations. Hakeberg and Berggren [8] assessed a Swedish language version of the DFS. These authors found a five-factor structure in exploratory factor analysis but reported a bad fit of this model in confirmatory factor analysis. They reported moderate correlation coefficients among the five factors.

Involving only parents or guardians, most of whom were female, in validity study may inflate the DFS scores over those that might be seen in the general population, since the females often report being more fearful than males [9-11]. However, it is not possible to judge any effect on the results. Future studies may be helpful in this regard.

## Conclusion

The Japanese version of the DFS appears reliable and demonstrates cross-cultural validity. The modeling confirms the three factors on which the English language version was based.

## Competing interests

The authors declare that they have no competing interests.

## Authors' contributions

TY participated in the design of the study, developed the instruments (made a backward translation of the Japanese version to English), performed statistical analysis, and drafted the manuscript. PM participated in the design of the study, in the analysis and interpretation of data, and in revising the manuscript. YM facilitated the participation of the schools and collected data. YN participated in the design of the study and developed the instruments (made a forward translation of the English version to Japanese). MK collected data and performed statistical analyses. TS participated in the design of the study. ANAD performed statistical analyses, interpreted the data and participated in revising the manuscript. All authors read and approved the final manuscript.

## Pre-publication history

The pre-publication history for this paper can be accessed here:



## Supplementary Material

Additional file 1 **The Japanese version of the DFS**. The file is the Japanese version of the DFS. Click here for file
