# Supplementary material for: Reliability and cross-cultural validity of a Japanese version of the Dental Fear Survey
Source: BMC Oral Health. 2009 Jul 10;9:17. doi: 10.1186/1472-6831-9-17 (PMC2718877; doi:10.1186/1472-6831-9-17)
Supplement: Additional file 1 — The Japanese version of the DFS. The file is the Japanese version of the DFS. [file 1472-6831-9-17-S1.pdf]

The Japanese version of the DFS

次の問は、歯科治療時において様々な状況・感情・反応を想定したものです。あなたの場合に最もあてはまる番号に○つけてください。

１．歯科治療に対する恐怖から、あなたは歯科医の予約をさき延ばしたことがありますか？

　１．全く無い　２．１回か２回ある　３．数回ある　４．しばしばある　５．いつもしている

２．歯科治療に対する恐怖から、あなたは予約をキャンセルしたり、また無断で予約をすっぽかしたりしたことがありますか？

　１．全く無い　２．１回か２回ある　３．数回ある　４．しばしばある　５．いつもしている

次の問３～９は、あなた自身が実際に歯科治療を受けていると想定して答えてください。

３．歯科治療時、あなたは体がかたくなりますか？

　１．全くならない　２．少しなる　３．幾分なる　４．かなりなる　５．非常になる

４．歯科治療時、あなたの呼吸は早くなりますか？

　１．全くならない　２．少しなる　３．幾分なる　４．かなりなる　５．非常になる

５．歯科治療時、あなたは汗をかきますか？

　１．全くかかない　２．少しかく　３．幾分かく　４．かなりかく　５．非常にかく

６．歯科治療時、あなたは気分が悪くなったり胃が痛くなったりしますか？

　１．全くならない　２．少しなる　３．幾分なる　４．かなりなる　５．非常になる

７．歯科治療時、あなたは心臓の鼓動が早く（ドキドキ）なりますか？

　１．全くならない　２．少しなる　３．幾分なる　４．かなりなる　５．非常になる

次の問８～20 は､多くの人が歯科に関して不安や恐怖心を感じる事柄や状況について述べてあります｡あなたは問８～20 の状況においてどの程度の不安や不快感をうけるでしょうか？あなたの場合に最もあてはまる番号に○をつけてください。（自分自身が、今その場面にいると想定してください。）

８．歯科で次回の予約をとっている時、不安を感じますか？

１．全く感じない　２．少し感じる　３．幾分感じる　４．かなり感じる　５．非常に感じる

９．歯科医院へ行く時、不安を感じますか？

１．全く感じない　２．少し感じる　３．幾分感じる　４．かなり感じる　５．非常に感じる

１０．待合室で待っている時、不安を感じますか？

１．全く感じない　２．少し感じる　３．幾分感じる　４．かなり感じる　５．非常に感じる

１１．治療台にすわった時、不安を感じますか？

１．全く感じない　２．少し感じる　３．幾分感じる　４．かなり感じる　５．非常に感じる

１２．歯科医院独特のにおいをかぐと、不安を感じますか？

１．全く感じない　２．少し感じる　３．幾分感じる　４．かなり感じる　５．非常に感じる

１３．歯科医師が近づいてきたのを見ると、不安を感じますか？

１．全く感じない　２．少し感じる　３．幾分感じる　４．かなり感じる　５．非常に感じる

１４．注射の針を見ると、不安を感じますか？

１．全く感じない　２．少し感じる　３．幾分感じる　４．かなり感じる　５．非常に感じる

１５．注射の針が刺入されたのを感じると、不安を感じますか？

１．全く感じない　２．少し感じる　３．幾分感じる　４．かなり感じる　５．非常に感じる

１６．歯を削る器械を見ると、不安を感じますか？

１．全く感じない　２．少し感じる　３．幾分感じる　４．かなり感じる　５．非常に感じる

１７．歯を削る器械の音を聞くと、不安を感じますか？

１．全く感じない　２．少し感じる　３．幾分感じる　４．かなり感じる　５．非常に感じる

１８．歯を削る器械の振動を感じると、不安を感じますか？

１．全く感じない　２．少し感じる　３．幾分感じる　４．かなり感じる　５．非常に感じる

１９．歯科医院に行って口の中をきれいに磨いてもらう時、不安を感じますか？

１．全く感じない　２．少し感じる　３．幾分感じる　４．かなり感じる　５．非常に感じる

２０．歯科治療全般について考えた時、不安を感じますか？

１．全く感じない　２．少し感じる　３．幾分感じる　４．かなり感じる　５．非常に感じる
